# Longitudinal Cognitive Diagnostic Assessment Based on the HMM/ANN Model

**DOI:** 10.3389/fpsyg.2020.02145

**Published:** 2020-09-09

**Authors:** Hongbo Wen, Yaping Liu, Ningning Zhao

**Affiliations:** ^1^National Assessment Center of Education Quality, Beijing Normal University, Beijing, China; ^2^School of Chinese Language and Literature, Beijing Normal University, Beijing, China

**Keywords:** cognitive diagnostic assessment, longitudinal assessment, hidden Markov model, SSOM neural network, reading comprehension

## Abstract

Cognitive diagnostic assessment (CDA) is able to obtain information regarding the student’s cognitive and knowledge development based on the psychometric model. Notably, most of previous studies use traditional cognitive diagnosis models (CDMs). This study aims to compare the traditional CDM and the longitudinal CDM, namely, the hidden Markov model (HMM)/artificial neural network (ANN) model. In this model, the ANN was applied as the measurement model of the HMM to realize the longitudinal tracking of students’ cognitive skills. This study also incorporates simulation as well as empirical studies. The results illustrate that the HMM/ANN model obtains high classification accuracy and a correct conversion rate when the number of attributes is small. The combination of ANN and HMM assists in effectively tracking the development of students’ cognitive skills in real educational situations. Moreover, the classification accuracy of the HMM/ANN model is affected by the quality of items, the number of items as well as by the number of attributes examined, but not by the sample size. The classification result and the correct transition probability of the HMM/ANN model were improved by increasing the item quality and the number of items along with decreasing the number of attributes.

## Introduction

Cognitive diagnostic assessment (CDA) combines cognitive psychology with psychometrics to diagnose and evaluate the knowledge structure and cognitive skills of students (Leighton and Gierl, 2007; Tu et al., 2012). Compared to the traditional academic proficiency assessment, the results of CDA report specific information regarding the strengths and the weaknesses of students’ cognitive skills. At present, researchers developed various cognitive diagnostic models (CDMs) to realize the diagnostic classification of cognitive skills. Deterministic inputs, noisy “and” gate (DINA) model (Macready and Dayton, 1977; Haertel, 1989; Junker and Sijtsma, 2001), the deterministic inputs, noisy “or” gate (DINO) model (Templin and Henson, 2006), and other models are representative and widely applied. However, traditional CDMs, such as DINA and DINO, are static models that classify students’ cognitive skills on a cross-sectional level. In the education context, students’ knowledge and skills are continually developing, and educators are more concerned with how their cognitive skills develop over time. Notably, traditional CDM cannot model the trajectory of skills development.

In the psychometric field, researchers have used multi-dimensional Item Response Theory (IRT) models to assess the development of students’ abilities (Andersen, 1985; Embretson, 1991). These studies utilized multi-dimensional IRT models to measure a single capability at different points in time. With the development of computer algorithms, the hidden Markov model (HMM) can be used to realize the transformation analysis of potential categories (Collins and Lanza, 2010). Currently, DINA and DINO models have been applied as measurement models under the framework of HMM (Li et al., 2016; Kaya and Leite, 2017). Chen et al. (2017) used the first-order HMM to trace learning trajectory. Additionally, Wang et al. (2018) integrated the CDM with a higher-order HMM, which included covariates, to model skill transition and explain individual differences.

This research mentioned above used HMM to realize the transformation analysis of potential states. The methods combine HMM and traditional CDMs, such as DINA and DINO, which are based on the framework of the IRT (Tu et al., 2012). As a result, the methods should satisfy the three basic hypotheses of unidimension, local independence, and monotonicity of capability. The problem is that the data collected in practice can hardly satisfy these three hypotheses. In recent years, researchers have attempted to develop more applicable models to new models to overcome these deficiencies. For instance, Hansen (2013) proposed a unidimensional hierarchical diagnostic model to track the growth of skill, in which local dependence was accounted for through using random-effect latent variables. Furthermore, Zhan et al. (2019) proposed a longitudinal diagnostic classification modeling approach by using a multidimensional higher-order latent structure to explain the relationship among multiple latent attributes, and the local item dependence was well taken into account.

The method mentioned above requires parameter estimation, which involves a large sample size to achieve high accuracy. When the sample size is small, the accuracy of parameter estimation will be affected (Chen et al., 2013), thus seriously influencing the accuracy of the cognitive skill classification of students (Gierl et al., 2008; Cao, 2009; Shu et al., 2013). Some researchers proposed to apply non-parametric methods to classify cognitive skills under a small sample size. For instance, Chiu et al. (2018) used general non-parametric classification method to estimate the student’s attribute pattern through minimizing the distance between the observed response and the ideal response when sample sizes are at the classroom level. With the development of artificial intelligence, ANN has been widely applied in various fields. And it is claimed that non-parametric artificial intelligence pattern recognition technology can be utilized to achieve CDA. The advantage is that ANN can perform non-parameter estimation and the bias of the potential classification model can be overcome (Gierl et al., 2008; Cao, 2009; Wang et al., 2015). Additionally, ANN has a relatively high accuracy in small samples, so it can avoid the above-mentioned disadvantages.

Recently, an increasing number of studies have attempted to combine ANNs and CDA (Gierl et al., 2008; Cao, 2009; Shu et al., 2013; Wang et al., 2015, 2016). At present, there are hundreds of artificial neural networks (ANN), among which the supervised self-organizing map (SSOM) is one of the more popular neural networks. SSOM has been widely used in network traffic classification, decoding analyses, and metabolic profiling and demonstrates good classification performance (Wongravee et al., 2010; Hu, 2011; Hu et al., 2011; Lu et al., 2020). The SSOM can activate the network features near the physical location of the neurons according to the similar input mode used to achieve classification, so it has a strong applicability in various fields. Consequently, it is worth further exploring whether it is possible to apply ANN (e.g., SSOM) as the measurement model of HMM so as to achieve the accurate classification of students’ cognitive skills while also tracking the development of their skills.

This study aims to explore whether it is possible to establish an HMM/ANN model through using ANN as the measurement model of HMM. This will be used to accurately track the change in students’ cognitive skills and to validate the effectiveness of this model in the actual education situation of the small sample.

## Technical Background

### An Overview of the Artificial Neural Network

Scientists Warren McCulloch and Walter Pitts first proposed the ANN in 1943, which mimics the basic principle of the biological nervous system. It is a network structure system created by a large number of interconnected neurons similar to the neurocyte in the human brain. In ANN, the neurons are usually organized into layers, such as the input layer and the output layer, and information processing is achieved through adjusting the connection between the nodes of each layer (Han, 2006).

The connection between the input and the output layers of ANN can be obtained through performing the training and the testing phases. In the training phase, the input data and the output data (or those only containing input data) of the training set will be applied to train the network. This is done to determine the number of hidden layer neurons as well as the connection weight between layers of neurons. Then, the neural network will be well trained. During the testing phase, a well-trained neural network will be provided a new set of input data that can obtain the output value based on the weight of connections between neurons.

According to the classification of learning paradigm, ANN can be divided into supervised learning and unsupervised learning. The most significant feature of supervised learning is that the data of the input and the output layer of the training set are known, and the output layer is the category label corresponding to the data characteristics of the input layer. The supervised neural network determines the connection weight of the layers through establishing the relationship between the input and the output layers. When there is new data input, the determined connection weights can assist in obtaining the output value. Notably, the characteristic of unsupervised learning is that only the data in the input layer is known, while the data in the output layer is unknown. Unsupervised neural network reveals the innate law of the data by learning the input data, which is more applicable to cluster analysis.

### Supervised Self-Organizing Map

**FIGURE 1:**
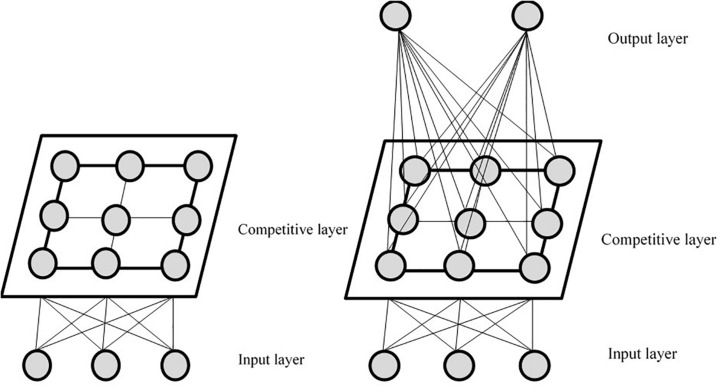
Basic structure of self-organizing map and supervised self-organizing map, respectively.

As depicted in Figure 1, the SSOM consists of three layers: the input layer, the competition layer, and the output layer. The number of output layers is consistent with the number of classification categories. SSOM is based on the original structure of the self-organizing mapping neural network (Kohonen, 1982, 1990, 2001), adding an output layer to become a supervised neural network, to better realize the classification of data with category labels. In SSOM, the part from the output layer to the competition layer is unsupervised learning, and the competition layer to the output layer is supervised learning. Moreover, the input layer to the competition layer and the competition layer to the output layer are all connected (Zhao and Li, 2012). Notably, it is necessary for the learning and the training of this neural network to adjust the weights from the input layer to the competition layer and from the competition layer to the output layer simultaneously. SSOM can use the existing category marker information to assist clustering and help improve the adjustment rules of neuron weight in the winning neighborhood. This is done so as to make it easier to select winning neurons (Hu et al., 2011).

In the training phase of SSOM, the input training samples *X*_*i*_ = (*X*_1_,*X*_2_,*X*_3_……*X*_*n*_) are known, and *n* is the number of neurons in the input layer. According to formula (1), the winning neuron *g* in the competitive layer can be obtained. *D*_*j*_ is the distance from input layer *X*_*i*_ to the neuron *j* in the competition layer, from which the winning neuron *g* with the smallest distance from the input layer *X*_*i*_ is found.

(1) $$D_{g}=\min\left(D_{j}\right)=\text{min}||X_{i}-W_{ij}||,j=1,2,\ldots ,m$$

Among them, ||⋅|| is the distance function; *W*_*ij*_ represents the weighting coefficient between the input layer neuron *i* and the competition layer neuron *j*; *m* is the number of competition layer neurons.

The next step is to adjust the weight, which is mainly divided into three phases:

(1) *Y*_*k*_ = (*Y*_1_,*Y*_2_,*Y*_3_……*Y*_*k*_) is the output value of the input layer *X*_*i*_, *k* represents the number of neurons in the output layer, while the output category corresponding to the winning neuron *g* in the competition layer is *O*_*g*_.
(2) Calculate the winning neighborhood *N*_*c(t)*_ of the winning neuron *g*.
(3) If *O*_*g*_ = *Y*_*i*_, then the weight coefficient is adjusted according to formula (2, 3) in the winning neighborhood; if *O*_*g*_≠*Y*_*k*_,then it is adjusted according to formula (4, 5).

(2) $$W_{ij}=W_{ij}+\eta_{1}\left(X_{i}-W_{ij}\right)$$

(3) $$W_{jk}=W_{jk}+\eta_{2}\left(Y_{k}-W_{jk}\right)$$

(4) $$W_{ij}=W_{ij}+\mu \eta_{1}\left(X_{i}-W_{ij}\right)$$

(5) $$W_{jk}=W_{jk}+\mu \eta_{2}\left(Y_{k}-W_{jk}\right)$$

*W*_*ij*_ represents the weight coefficient between the input layer neuron *i* and the competition layer neuron *j*; *W*_*jk*_ represents the weight coefficient between the competition layer neuron *j* and the output layer neuron *k*; η_*1*_, η_*2*_ represents the learning rate from the input layer to the competition layer and from the competition layer to the output layer, respectively; μ is the weight coefficient. After adjusting the weights, the output layer becomes an ordered feature graph which reflects the output pattern.

### Hidden Markov Model

**FIGURE 2:**
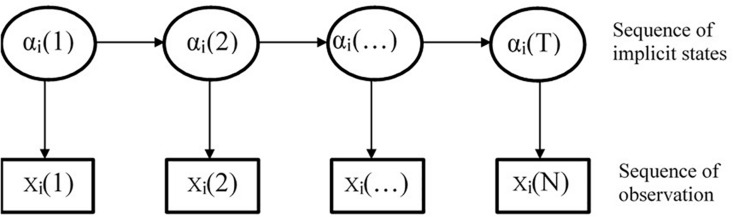
Hidden Markov model.

The HMM is also known as the potential transformation analysis model (Collins and Wugalter, 1992). As depicted in Figure 2, the model contains two interconnected random processes. One describes a Markov chain of state transition and the other is a sequence of observations related to states. The reason why it is referred to as the HMM is because, in these two random processes, the first random process, namely, the sequence of state transition, is unobserved and can only be inferred from the observation sequence of the other random process (Rabiner, 1989).

HMM can be described by five parameters:

*N*: *N* is the number of states of the Markov chain in the model. The *N* state is denoted as *S* = {*s*_1_,*s*_2_, *s*_3_…*s*_*N*_}, and the state of the Markov chain at time *t* is *q*_*t*_, *q*_*t*_ ∈ (*s*_1_,*s*_2_, *s*_3_…*s*_*N*_).
*M: M* is the number of possible observations for each state. *M* observations are denoted as *V* = {*v*_1_,*v*_2_, *v*_3_…*v*_*M*_}, and the observed value at time *t* is *O*_*t*_, *O*_*t*_ ∈ (*v*_1_,*v*_2_, *v*_3_…*v*_*N*_).

π: π is initial-state probability,π = (π_*i*_,*i* = 1,…,*N*).

(6) $$\pi_{i}=P\left(q_{1}={\text{s}}_{i}\right)$$

(7) $$0\leq \pi_{i}\leq 1,\sum_{i=1}^{N}\pi_{i}=1\;$$

*A: A* is the state transition probability matrix (*a*_*ij*_)_*N ×N*_, which describes the state transition probability at different points in time. Among them:

(8) $$a_{ij}=P\left(q_{t}={\text{s}}_{j}|q_{t-1}={\text{s}}_{i}\right),1\leq \text{i}\leq N$$

$$0\leq a_{ij}\leq 1,\sum_{j=1}^{N}a_{ij}=1\;$$

*B: B* is an observation probability matrix, namely, the item response probability matrix (*b*_*jk*_)_*N ×N*_. In the educational measurement field, it refers to the probability that the individual in each potential state makes a correct or specific response to each item. Among them:

(9) $$b_{jk}=P\left(O_{t}=v_{k}|q_{\text{t}}={\text{s}}_{j}\right)$$

(10) 1≤j≤N,1≤k≤M

(11) $$0\leq b_{jk}\leq 1,\sum_{k=1}^{N}b_{jk}=1$$

That is, in state *j*, the probability that the observation is *k*.

In general, HMM consists of two parts. One of which is a Markov chain (namely, the transition model), which is utilized to describe the change of the hidden state. It is described by the initial state π and the transition probability matrix *A*, and different π and *A* determine the different topological structures of the Markov chain and affect the complexity of the model. The other part is the measurement model (namely, the observation probability), which is determined by the observation probability matrix *B*, connecting the observation score and the hidden state.

### HMM/ANN Model

HMM is composed of two parts: the Markov chain and the measurement model. It has a very strong modeling capability of dynamic temporal sequence, which can help us solve issues in timing changes and provide an excellent theoretical framework for realizing the longitudinal tracking of cognitive skills. However, HMM does not have a strong classification ability and cannot be directly used for longitudinal CDA as the measurement model in HMM is not suitable for cognitive diagnosis analysis. The observed probability in HMM represents the probability that the individual in each potential state makes a correct or specific response to each item. HMM can be regarded as an exploratory method to mark the potential state which is based on the item response probability (Nylund et al., 2007). However, CDA is different. This is because the categories of attributes or attribute mastery patterns are known in CDA, and it is necessary to obtain the probability of each category, which is a type of confirmatory process. ANN, on the contrary, has a strong classification ability, which can make up for this shortcoming. Considering the respective superiority of HMM and ANN, it is worth further exploring whether the HMM and the ANN can be combined to realize longitudinal CDA.

**FIGURE 3:**
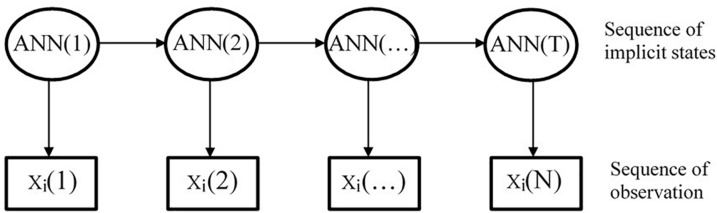
Hidden Markov model/artificial neural network model.

At present, there are several ways to combine HMM with ANN, one of which is to calculate the observation probability of the HMM through the ANN model, taking ANN as the measuring model of HMM (Bourlard and Morgan, 1997), as shown in Figure 3. The concrete implementation method is to use ANN model to calculate the observation probability of HMM, that is, to calculate the observation sequence probability value under each state through the ANN model. Then, the transformation probability of the observation sequence is obtained through the Markov chain in HMM.

## Simulation Study

### Research Question

The simulation study includes the following question:

Can the HMM/ANN model accurately track the development of students’ cognitive skills?

### Method

#### Data Generation

This study simulates longitudinal data with three time points (T1/T2/T3) based on the DINA model, and several key factors were manipulated, including the number of items (20 or 40), the number of attributes (3 or 6), sample size (200, 500, or 1,000) as well as item discrimination (high or mixed). High item discrimination indicates smaller slip and guess parameters, which are randomly generated on the uniform distribution *U*(0, 0.20). The mixed discriminability contains both small and large slip and guess parameters, which were randomly generated based on the uniform distribution *U*(0, 0.40). The selection of these factor levels is based on typical settings in recent simulation studies (e.g., Henson and Douglas, 2005; Rupp and Templin, 2008; Templin et al., 2008; de La Torre and Lee, 2010; Cui et al., 2016). Notably, based on the item and attribute level, four *Q* matrices were established. The *Q* matrices, along with item parameters for 20 items, are presented in Table 1.

**TABLE 1:** Q matrices and item parameters used in the simulation (20 items).

Item				Discrimination										Discrimination			
	*k* = 3			High		Mixed		*k* = 6						High		Mixed	
	A1	A2	A3	s	g	s	g	A1	A2	A3	A4	A5	A6	s	g	s	g
1	1	1	0	0.16	0.04	0.33	0.1	1	0	1	1	0	0	0.08	0.17	0.4	0.35
2	0	1	1	0.17	0.15	0.24	0.28	1	1	0	0	0	1	0	0.05	0.35	0.09
3	1	1	0	0.01	0.1	0.26	0.24	1	0	1	1	0	1	0.03	0.15	0.21	0.22
4	1	1	1	0.17	0.05	0.12	0.22	0	1	0	1	1	0	0.11	0.05	0.4	0.21
5	0	1	1	0.1	0.13	0.37	0.15	1	0	0	0	1	0	0.03	0.14	0.39	0.03
6	0	1	0	0.12	0.07	0.35	0.04	0	0	1	0	0	1	0.03	0.13	0.02	0.02
7	1	0	0	0.07	0.15	0.09	0.34	1	1	0	1	0	1	0.19	0.02	0.31	0.16
8	1	0	1	0.08	0.17	0.39	0.14	1	1	1	1	1	0	0.18	0.19	0.03	0.14
9	0	1	0	0.03	0.1	0.19	0.36	1	0	0	1	0	0	0.07	0.06	0.28	0.16
10	0	1	1	0.16	0.07	0.31	0.3	1	1	0	0	0	0	0.03	0.19	0.3	0
11	1	0	1	0.1	0.17	0.11	0.38	0	0	0	1	0	0	0.08	0.16	0.06	0.05
12	0	1	0	0.16	0.11	0.3	0.04	0	0	0	1	0	0	0.06	0.05	0.11	0.32
13	1	0	1	0.13	0.1	0.33	0.19	1	0	0	1	0	0	0	0	0.32	0.21
14	0	1	1	0.03	0.13	0.09	0.18	0	1	0	0	1	1	0.12	0.04	0.33	0
15	1	0	1	0.05	0.17	0.37	0.39	1	1	1	1	1	1	0.05	0.04	0.08	0.11
16	1	1	0	0.05	0.01	0.29	0.06	0	1	0	1	1	1	0.15	0.06	0.12	0.12
17	1	0	1	0.14	0.05	0.39	0.1	0	0	1	0	0	0	0.05	0.16	0.32	0.09
18	1	0	0	0.1	0.17	0.31	0.01	1	1	0	0	0	1	0.07	0.01	0.22	0.04
19	0	1	0	0.11	0.03	0.12	0.19	1	0	0	0	0	1	0.06	0.05	0.32	0.05
20	0	1	1	0.14	0.18	0.18	0.06	1	1	1	1	0	1	0.2	0.04	0.14	0.23

To evaluate whether the HMM/ANN model can accurately track the development of students’ cognitive skills, this study fixes the initial mastery probability as well as the transition probability of each attribute. Through combining these two probabilities, the attribute mastery probability and the increase of the attribute mastery probability at time points T2 and T3 can be obtained. Based on previous studies (Madison and Bradshaw, 2018) on the setting of the initial attribute mastery probability, this study sets the initial attribute mastery probability as 0.4, 0.4, and 0.2 under the condition of three attributes. Notably, this study assumes that it is unlikely that students’ mastery of the first two attributes will decrease in a relatively short teaching period, while the mastery of the third attribute may decline. Consequently, the transition probability of attribute loss is 0.1, 0.08, and 0.38, respectively. Under the condition of six attributes, the initial attribute mastery probability is 0.4, 0.4, 0.3, 0.3, 0.2, and 0.2. Meanwhile, the attribute loss transfer probability is 0.03, 0.04, 0.13, 0.25, 0.43, and 0.54, respectively. The correlation coefficient between the attributes at the initial time point is fixed at 0.5.

Table 2 depicts the transition probability matrix of each attribute under the conditions of the three and six attributes. In the matrix, 0 indicates that the student did not master the attribute, whereas 1 indicates that the student mastered the attribute. The matrix (from left to right) reflects the probability of students moving from non-mastery to non-mastery, from non-mastery to mastery, from mastery to non-mastery, and from mastery to mastery. Table 3 illustrates the probability and the growth rate of students’ attribute mastery at each time point.

**TABLE 2:** Conditional transition probability.

Number of attributes	Attribute	T1–T2			T2–T3		
			0	1		0	1
	A1	0	0.55	0.45	0	0.66	0.34
3		1	0.03	0.97	1	0.06	0.94
	A2	0	0.71	0.29	0	0.82	0.18
		1	0.03	0.97	1	0.05	0.95
	A3	0	0.93	0.07	0	0.9	0.1
		1	0.87	0.13	1	0.54	0.46
	A1	0	0.45	0.55	0	0.36	0.64
		1	0.03	0.97	1	0.02	0.98
	A2	0	0.56	0.44	0	0.42	0.58
		1	0.04	0.96	1	0.05	0.95
6	A3	0	0.61	0.39	0	0.54	0.46
		1	0.13	0.87	1	0.14	0.86
	A4	0	0.72	0.28	0	0.46	0.54
		1	0.25	0.75	1	0.06	0.94
	A5	0	0.78	0.22	0	0.42	0.58
		1	0.43	0.57	1	0.18	0.82
	A6	0	0.83	0.17	0	0.59	0.41
		1	0.54	0.46	1	0.12	0.88

**TABLE 3:** Attribute mastery probability and growth rate.

Number of attributes	Attribute		T1 (%)	T2 (%)	Growth rate	T3 (%)	Growth rate
3	A1	Non-mastery probability	0.6	0.37	0.23	0.23	0.14
		Mastery probability	0.4	0.63		0.77	
	A2	Non-mastery probability	0.6	0.46	0.14	0.34	0.11
		Mastery probability	0.4	0.54		0.66	
	A3	Non-mastery probability	0.8	0.7	0.1	0.62	0.08
		Mastery probability	0.2	0.3		0.38	
6	A1	Non-mastery probability	0.6	0.34	0.26	0.15	0.20
		Mastery probability	0.4	0.66		0.85	
	A2	Non-mastery probability	0.6	0.35	0.25	0.20	0.15
		Mastery probability	0.4	0.65		0.80	
	A3	Non-mastery probability	0.7	0.51	0.19	0.39	0.12
		Mastery probability	0.3	0.49		0.61	
	A4	Non-mastery probability	0.7	0.57	0.13	0.45	0.11
		Mastery probability	0.3	0.43		0.55	
	A5	Non-mastery probability	0.8	0.68	0.12	0.55	0.13
		Mastery probability	0.2	0.32		0.45	
	A6	Non-mastery probability	0.8	0.74	0.06	0.64	0.10
		Mastery probability	0.2	0.26		0.36	

To simulate the observed item response, the students’ “true” attribute pattern must be generated. In order to ensure an equal representation of the different attribute patterns, we assumed that student attribute patterns satisfy a uniform distribution. According to the students’ “true” attribute pattern, *Q* matrix, item slid and guess parameter, and students’ responses to each item were simulated. To train the neural network, input and output data are essential, that is, students’ “true” attribute patterns and their response to items are required, which cannot be obtained through practice. Therefore, the ideal response, the ideal response vector, and its related true attribute pattern are utilized to train the neutral network. Furthermore, since we set the transition probability of each attribute, our simulated data can reflect the growth of cognitive skills. There are 2 × 2 × 3 × 2 = 24 conditions for each point in time. To obtain stable simulation results, each condition was repeated 30 times. Specifically, the R.3.1.0 (R Development Core Team, 2006) software CDM package was applied to generate data.

#### SSOM for CDA

The SSOM was used to classify the simulated item response. SSOM is comprised of three layers: the input layer, the competition layer, and the output layer. The number of nodes in the input layer of SSOM is the number of items (20 or 40), and the input data is the students’ response to each item. The number of nodes in the output layer represents the number of attribute mastery pattern categories, and three or six skills correspond to the 2^3^ = 8 or 2^6^ = 64 attribute mastery pattern, and the data in the output layer is the attribute mastery pattern. Notably, there are two phases to implement SSOM to estimate the attribute patterns of the simulated item response:

Step 1: Training phase

This study simulates the data of the training set, applying the simulated ideal item response as the input value of the training set and the true attribute pattern as the output value of the training set. The input and the output layers are known, so only the number of neurons in the competing layer needs to be determined. Cui et al. (2016) empirically suggested that the number of nodes in the competing layer should be set to four to 10 times the number of attribute mastery patterns. This study conducted experiments on the number of neurons in the competitive layer under the conditions of three and six attributes and discovered that the number of neurons had no profound impact on the classification accuracy. Consequently, the structure of the neurons in the competitive layer was finally set to 10^∗^10 and 20^∗^20 under the conditions of three and six attributes, respectively.

Moreover, the classification accuracy of SSOM is greatly influenced by the number of iterations. The more iterations, the higher the classification accuracy. As the number of iterations increases, however, so does the elapsed time. Because of this, it is necessary to determine the appropriate number of iterations. This study further explored the classification accuracy of SSOM under different iterations through experiments to determine the iterations. Firstly, the number of iterations was set to 1, and the classification accuracy of the training set was recorded. Then, the number of iterations was increased one by one and the process was repeated until the accuracy of the training set became stable. The accuracy of the training set was stable after two iterations under the condition of three attributes, and the accuracy was 99.5% and 100% for 20 and 40 items. As a result, the number of iterations under this condition is determined to be two. Additionally, the accuracy of the training set became stable after four and seven iterations under the condition of six attributes, 20 and 40 items.

Step 2: Testing phase

After determining the structure and the number of iterations of the SSOM and training the neural network, the well-trained network can perform the diagnostic classification of cognitive skills on the simulated observed item response. If the attribute mastery patterns of the simulation data are estimated, the attribute accuracy rate (ACCR) and the pattern accuracy rate (PCCR) will be calculated through comparing the “true” and the estimated attribute mastery pattern. These two indicators were used as the primary criteria to evaluate the classification accuracy of SSOM. The training and the testing of SSOM were implemented through using the PyCharm software.

#### The Implementation of the HMM/ANN Model

In this study, the HMM is taken as the overall model, in which SSOM is used for the measurement model to realize the classification of the item response at each time point, while the transition model part is a Markov model. We actually performed two steps to complete the entire model:

Step 1: SSOM is used to calculate the observation probability of HMM

Based on the two phases mentioned in “SSOM for CDA” the SSOM model is used to calculate the probability of the observation sequence in each state, that is, to obtain the information of the attribute mastery pattern at each time point. This is actually the completion of the measurement model of HMM.

Step 2: Calculate the transition probability for HMM

Then, the Markov model in HMM was implemented to obtain information of students’ attribute growth. The transition probability of the attribute mastery pattern between time points was calculated by applying the Markov chain. Meanwhile, Matlab was used to calculate the transformation probability. By comparing the true value and the estimated value of attribute transfer probability, the average correct transformation rates of the HMM/ANN model was evaluated.

### Results

Table 4 presents the classification accuracy of SSOM under the three attributes at each time point. Notably, the number of items has a positive influence on the classification accuracy—the larger the number of items, the higher the classification accuracy of SSOM. For instance, compared with 20 items, the classification accuracy of the attribute mastery pattern by SSOM increased from 0.91 to 0.97 under the condition of high discrimination, 500 samples, and 40 items. Furthermore, the discrimination has a positive influence on the classification accuracy. With the decrease of item discrimination, the classification accuracy of each attribute and the attribute mastery pattern also decrease. For instance, the classification accuracy of the attribute pattern by SSOM is between 0.91 and 0.92 under the condition of 500 samples and high discrimination. Meanwhile, in the case of mixed discrimination, the classification accuracy is between 0.74 and 0.80. This is consistent with our expectations, and items with low discrimination are difficult to distinguish as to whether students have mastered or not. Additionally, the influence of sample size is relatively small or absent, with the other conditions unchanged. The classification accuracy of the attribute pattern is between 0.84 and 0.91 under the condition of 200 samples, 20 items, and high discrimination. Moreover, the classification accuracy of the attribute pattern is between 0.91 and 0.92, under the same condition of 500 and 1,000 samples. It can be seen that, under the condition of 200 samples and 20 items, the classification accuracy of SSOM is slightly lower than that of 500 or 1,000. Meanwhile, under the condition of 200 samples, 40 items, and high discrimination, the classification accuracy of the attribute pattern is between 0.94 and 0.97, which is very close to the classification accuracy under the condition of sample size 500 and 1,000. Generally, SSOM has a slightly different classification accuracy under the sample size of 200, 500, and 1,000, but its classification accuracy is generally relatively consistent.

**TABLE 4:** Attribute classification accuracy (*k* = 3).

Sample size	Number of items	Item discrimination	Time point	Classification accuracy			
				A1	A2	A3	Attribute mastery pattern
200	20	High (0, 0.2)	T1	0.9	0.97	0.96	0.84
			T2	0.93	0.98	0.98	0.87
			T3	0.95	0.98	0.98	0.91
		Mixed (0, 0.4)	T1	0.84	0.89	0.9	0.75
			T2	0.88	0.92	0.92	0.74
			T3	0.91	0.94	0.94	0.79
	40	High (0, 0.2)	T1	0.99	0.98	0.98	0.97
			T2	0.98	0.98	0.99	0.94
			T3	0.98	0.98	0.99	0.94
		Mixed (0, 0.4)	T1	0.93	0.88	0.92	0.75
			T2	0.89	0.84	0.93	0.68
			T3	0.87	0.84	0.94	0.68
500	20	High (0, 0.2)	T1	0.94	0.98	0.98	0.91
			T2	0.94	0.98	0.99	0.92
			T3	0.94	0.98	0.99	0.91
		Mixed (0, 0.4)	T1	0.8	0.89	0.93	0.74
			T2	0.8	0.91	0.94	0.78
			T3	0.8	0.92	0.93	0.8
	40	High (0, 0.2)	T1	0.99	0.99	0.99	0.97
			T2	0.99	0.99	0.99	0.96
			T3	0.99	0.99	0.99	0.98
		Mixed (0, 0.4)	T1	0.89	0.91	0.91	0.71
			T2	0.84	0.93	0.9	0.69
			T3	0.82	0.94	0.9	0.69
1,000	20	High (0, 0.2)	T1	0.94	0.97	0.99	0.91
			T2	0.94	0.98	0.99	0.92
			T3	0.93	0.98	0.98	0.92
		Mixed (0, 0.4)	T1	0.8	0.91	0.94	0.68
			T2	0.81	0.91	0.93	0.65
			T3	0.82	0.92	0.92	0.61
	40	High (0, 0.2)	T1	0.99	0.99	0.99	0.99
			T2	0.99	0.99	0.99	0.99
			T3	0.99	0.99	1	0.99
		Mixed (0, 0.4)	T1	0.87	0.9	0.89	0.71
			T2	0.83	0.92	0.89	0.71
			T3	0.82	0.93	0.91	0.76

Table 5 illustrates the classification accuracy of SSOM under the six-attributes condition. Through comparing Tables 4, 5, it can be seen that when the number of attributes increased from three to six, the classification accuracy of SSOM decreases sharply. For instance, the classification accuracy of the attribute mastery pattern at the first time point is 0.91 under the condition of three attributes, 20 items, 500 samples, and a high degree of discrimination. Under the same condition, however, when the number of attributes is six, the classification accuracy of the attribute mastery pattern is 0.65. This is consistent with previous studies (Cui et al., 2016). When the number of attributes increases, the classification accuracy of both ANN and traditional CDM is poor. Moreover, the influence of sample size, number of items, and item discrimination is consistent with the results under the condition of three attributes, which will not be repeated here.

**TABLE 5:** Attribute classification accuracy (*k* = 6).

Sample size	Number of items	Item discrimination	Time point	Classification accuracy						
				A1	A2	A3	A4	A5	A6	Attribute mastery pattern
200	20	High (0, 0.2)	T1	0.74	0.71	0.74	0.85	0.82	0.90	0.45
			T2	0.78	0.78	0.79	0.86	0.80	0.62	0.37
			T3	0.86	0.89	0.83	0.88	0.80	0.83	0.56
		Mixed (0, 0.4)	T1	0.64	0.61	0.64	0.72	0.68	0.80	0.35
			T2	0.73	0.73	0.70	0.75	0.71	0.52	0.31
			T3	0.77	0.82	0.76	0.79	0.73	0.71	0.44
	40	High (0, 0.2)	T1	0.71	0.74	0.81	0.81	0.79	0.86	0.43
			T2	0.86	0.87	0.82	0.89	0.90	0.90	0.63
			T3	0.91	0.93	0.89	0.92	0.93	0.96	0.71
		Mixed (0, 0.4)	T1	0.59	0.62	0.74	0.70	0.66	0.81	0.33
			T2	0.76	0.77	0.73	0.79	0.74	0.78	0.39
			T3	0.86	0.85	0.78	0.84	0.76	0.85	0.57
500	20	High (0, 0.2)	T1	0.89	0.87	0.93	0.95	0.98	0.85	0.65
			T2	0.87	0.88	0.93	0.94	0.93	0.88	0.76
			T3	0.9	0.91	0.94	0.94	0.94	0.82	0.72
		Mixed (0, 0.4)	T1	0.77	0.8	0.84	0.8	0.81	0.74	0.36
			T2	0.77	0.8	0.83	0.8	0.82	0.74	0.37
			T3	0.84	0.84	0.86	0.81	0.83	0.71	0.36
	40	High (0, 0.2)	T1	0.88	0.9	0.82	0.92	0.94	0.92	0.7
			T2	0.89	0.92	0.87	0.91	0.94	0.92	0.75
			T3	0.89	0.91	0.9	0.91	0.94	0.94	0.73
		Mixed (0, 0.4)	T1	0.77	0.8	0.74	0.82	0.77	0.8	0.44
			T2	0.79	0.83	0.77	0.78	0.83	0.84	0.45
			T3	0.83	0.84	0.8	0.85	0.78	0.86	0.49
1,000	20	High (0, 0.2)	T1	0.87	0.89	0.91	0.94	0.93	0.88	0.58
			T2	0.87	0.88	0.91	0.94	0.93	0.86	0.56
			T3	0.9	0.92	0.93	0.94	0.93	0.83	0.64
		Mixed (0, 0.4)	T1	0.81	0.77	0.73	0.82	0.83	0.87	0.36
			T2	0.78	0.81	0.81	0.82	0.84	0.74	0.35
			T3	0.86	0.86	0.84	0.81	0.84	0.71	0.39
	40	High (0, 0.2)	T1	0.88	0.9	0.84	0.92	0.92	0.91	0.65
			T2	0.89	0.91	0.85	0.91	0.94	0.93	0.65
			T3	0.89	0.92	0.89	0.91	0.94	0.95	0.68
		Mixed (0, 0.4)	T1	0.84	0.85	0.77	0.85	0.75	0.85	0.45
			T2	0.79	0.82	0.76	0.79	0.82	0.85	0.44
			T3	0.85	0.86	0.81	0.81	0.82	0.89	0.52

Table 6 depicts the correct transition rate of the attribute mastering patterns obtained through the HMM/ANN model under each simulation condition. Under the condition of three attributes, the HMM/ANN model demonstrates a high correct transition rate from time 1 to time 2 and time 2 to time 3. The discrimination also has a positive influence on the correct transition rate. Notably, the correct transition rate is higher under the high-discrimination condition than in the mixed-discrimination condition. Under the condition of high discrimination, the HMM/ANN model has a high correct transition rate, which is 0.95–0.99. Under the mixed-discrimination condition, the correct transition rate is reduced, which is 0.87–0.95. The influence of the number of items on the HMM/ANN model is not clear in this simulation study. For example, under the condition of 20 items, the correct transition rate of the HMM/ANN model is 0.90–0.98. Meanwhile, under the condition of 40 items, the correct transition rate was 0.87–0.99. Additionally, the correct transition rate of the HMM/ANN model was also unaffected by the sample size.

**TABLE 6:** Correct transition rate of attribute master pattern.

Number of attributes	Sample size	Number of items	Item discrimination	Correct transition rate	
				T1–T2	T2–T3
3	200	20	High	0.95	0.96
			Mixed	0.91	0.92
		40	High	0.98	0.99
			Mixed	0.91	0.93
	500	20	High	0.98	0.97
			Mixed	0.92	0.9
		40	High	0.98	0.98
			Mixed	0.9	0.87
	1,000	20	High	0.98	0.98
			Mixed	0.94	0.95
		40	High	0.99	0.99
			Mixed	0.92	0.94
6	200	20	High	0.98	0.97
			Mixed	0.98	0.98
		40	High	0.99	0.99
			Mixed	0.98	0.97
	500	20	High	0.98	0.97
			Mixed	0.97	0.97
		40	High	0.98	0.99
			Mixed	0.97	0.98
	1,000	20	High	0.98	0.97
			Mixed	0.97	0.96
		40	High	0.98	0.99
			Mixed	0.98	0.98

Under the condition of six attributes, the correct transition rate of the HMM/ANN model is at a high level, ranging from 0.97 to 0.99, and it was difficult to identify the influence of sample size, the number of items, and the quality of items.

Even in the case of six attributes, the classification accuracy of ANN is reduced, but it does not affect the correct transition rate of the longitudinal model. This may be attributed to the fact that when six attributes are examined, there will be 2^6^ = 64 attribute mastery patterns, forming a 64^∗^64 transfer probability matrix, which is too large, thus affecting the calculation of the correct transition rate.

## Empirical Study

### Research Question

The empirical study includes the following question:

What is the effectiveness of the HMM/ANN model in real situations through tracking students’ mastery and development of cognitive skills based on actual reading literacy assessment data?

### Method

The empirical study analyzed the data of a reading literacy assessment completed by a school in Beijing. There were 190 students who completed the same reading passage—*book* in grade 4 (2015) and grade 5 (2016), which contains a total of eight items. All eight items are scored 0 (incorrect) or 1 (correct). The selected short test examines three skills of acquisition, integration, and evaluation and are examined by two, three, and three questions. The skills examined by each item are displayed in Table 7.

**TABLE 7:** The Q matrix of empirical data.

Item	Acquisition	Integration	Evaluation
1	1	0	0
2	1	0	0
3	0	1	0
4	0	1	0
5	0	0	1
6	0	0	1
7	0	0	1
8	0	1	0

The quality of the eight items is good. In terms of the fourth-grade test, the items have medium discriminations between 0.31 and 0.46, except for item 6 which has low discrimination of 0.21. For the fifth-grade test, the discrimination is lower than 0.3 (0.28 and 0.29), except for items 1 and 6. The other items have medium discrimination between 0.31 and 0.43.

The three-layer SSOM network structure was selected in the empirical study. Training of the neural network needs to include both input and output data. In the empirical study, however, we only have the input data in the testing set, namely, the observed item responses of students. To train the SSOM, it is necessary to have both input and output data, that is, the true attribute patterns of students and their response to items, which cannot be obtained in empirical data. Drawing from previous experience, we simulated the ideal item response and true attribute patterns based on the *Q* matrix of empirical data, which are used as the input and the output data of the training set. R.3.1.0 (R Development Core Team, 2006) CDM package was used to generate the training set data, and then PyCharm was used to train and test the SSOM. The determination of the number of nodes in the SSOM competition layer and the number of iterations is the same as in the simulation study. Finally, the network structure of the competition layer was set to 9^∗^9, and the number of iterations was determined to be three. Afterward, the observed responses were classified by the well-trained neural network. Similar to the simulation study, ACCR and PCCR were used as the main criteria to evaluate the classification accuracy of SSOM. As mentioned earlier, we simulated the ideal item response and the true attribute mastery patterns based on the *Q* matrix of the empirical data, so ACCR and PCCR can be successfully calculated by comparing the true and the estimated attribute mastery patterns. Then, Matlab was applied to calculate the transformation probability matrix.

### Results

Table 8 reflects the classification accuracy of SSOM for the three attributes examined in the fourth- and the fifth-grade tests as 0.97, 0.98, and 0.90 and 0.98, 0.95, and 0.91, respectively. The classification accuracy of the attribute master pattern is 0.87and 0.85, respectively. Notably, the results of the empirical study are similar to those of the simulation study. SSOM provided an accurate classification at two time points when the tests examined fewer skills and the quality of items was higher.

**TABLE 8:** The classification accuracy of supervised self-organizing map.

	Acquisition	Integration	Evaluation	Attribute mastery pattern
Fourth grade	0.98	0.99	0.91	0.87
Fifth grade	0.98	0.95	0.91	0.85

**FIGURE 4:**
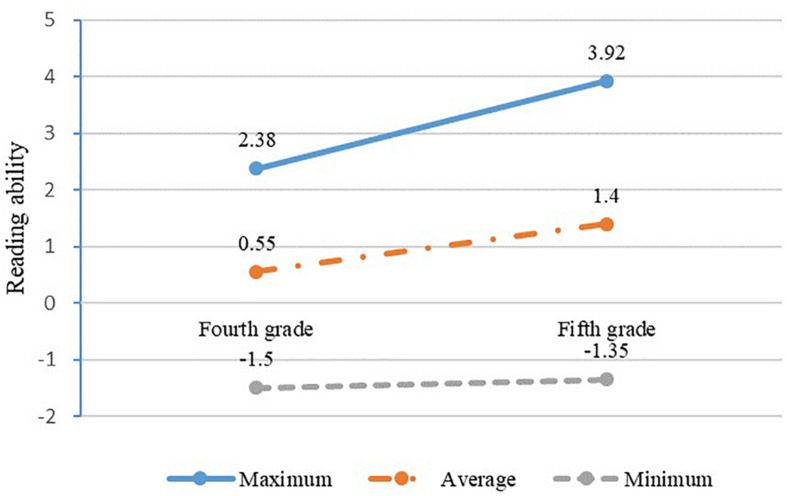
The change in students’ reading ability.

The development of students’ reading ability with time is displayed in Figure 4. The reading ability of students has improved during year 1. For example, the average reading ability increased from 0.55 to 1.40 from the fourth to the fifth grade.

**FIGURE 5:**
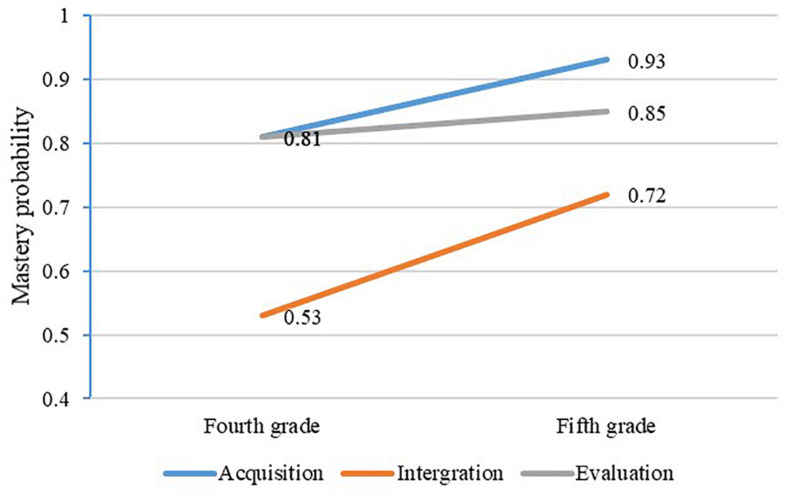
The change of attribute mastery probability.

Figure 5 illustrates the mastery of each attribute in grades four and five. In total, the mastery probability of these three attributes increases with time. For fourth-grade students, the attribute mastery probability is between 0.53 and 0.81, and the average mastery probability is 0.72. The attribute mastery probability is between 0.72 and 0.93 for fifth-grade students, and the average mastery probability is 0.68. Moreover, it can be observed that the mastery probability of acquisition and integration demonstrates the same growth trend. Additionally, the growth trend of evaluation is flatter, and the growth range of the three attributes is between 0.04 and 0.19.

Table 9 depicts the transformation probability matrix of each attribute. The four cells in each 2^∗^2 matrix represent (from left to right) non-mastery to non-mastery, non-mastery to mastery, mastery to non-mastery, as well as mastery to mastery. For the attributes of acquisition, integration, and evaluation, the probability from non-mastery to mastery is 0.80, 0.66, and 0.71, and the probability from mastery to non-mastery is 0.05, 0.19, and 0.14, respectively. This suggests that the majority of students can achieve the transition from non-mastery to mastery at the two time points, and a small percentage of students return from mastery to non-mastery.

**TABLE 9:** The transition probability matrix of attributes.

Attribute	Transition matrix			
**Acquisition**		**Fifth grade**		
			**0**	**1**

	Fourth grade	0	0.20	0.80
		1	0.05	0.95

**Integration**		**Fifth grade**		
			**0**	**1**

	Fourth grade	0	0.34	0.66
		1	0.19	0.81

**Evaluation**		**Fifth grade**		
			**0**	**1**

	Fourth grade	0	0.29	0.71
		1	0.14	0.86

Table 10 illustrates the transformation probability matrix of eight attribute mastery patterns. It can be seen that, among students who did not master any skills (000) in the fourth grade, there were still 25% of students who did not even master three skills in the fifth grade, and 38% of the students were able to master all three skills in the fifth grade, which shows a dramatic improvement. For students who fully mastered the three skills (111) in the fourth grade, 71% were still able to master the three skills in the fifth grade. For other categories of attribute mastery patterns, 40–63% of students were able to master all skills in the fifth grade.

**TABLE 10:** Transition probability matrix of attribute mastery pattern.

		T2 (fifth grade)							
		000	001	010	100	110	011	101	111
T1 (fourth grade)	000	0.25	0	0.13	0.13	0	0.13	0	0.38
	001	0	0.04	0.04	0.22	0	0	0.13	0.57
	010	0	0	0.13	0	0.13	0.13	0	0.63
	100	0	0.07	0	0	0.13	0	0.33	0.47
	110	0	0	0	0	0.3	0	0.3	0.4
	011	0	0	0.13	0	0.13	0.13	0	0.63
	101	0	0.02	0.02	0.02	0.12	0	0.24	0.56
	111	0	0.02	0.01	0.01	0.06	0.02	0.16	0.71

Longitudinal CDA can also assist in obtaining information regarding individuals. For example, the student with ID 3410105 scored 0.25 on average on eight items in the fourth grade, and their attribute mastery pattern was “100”. In the fifth grade, they scored 0.75, and their attribute mastery pattern was “111,” which means that they mastered all three skills. It can be seen that, after a year’s study, the students’ reading skills have significantly improved and they are able to master integration and evaluation skills. For the student with ID 3430308, they scored 0.38 on average on eight items in the fourth grade, and their attribute mastery pattern was “100”. When the student was in the fifth grade, they scored 0.50, but their attribute mastery pattern was also “100”. Which indicates that they had not mastered integration and evaluation skills.

## Discussion

### HMM/ANN Model Achieved Fine-Grained Longitudinal Tracking of Students’ Cognitive Skills

Previously, researchers tracked the development of cognitive skills in three ways. Firstly, they used the Multidimensional Item Response Theory (MIRT; Andersen, 1985; Embretson, 1991) to track changes in students’ abilities. The second was to integrate traditional CDMs, such as the DINA and the DINO models, within the framework of HMM (Li et al., 2016; Kaya and Leite, 2017; Madison and Bradshaw, 2018; Hung and Huang, 2019). The third was to construct higher-order latent structures for measuring growth to explain the relationship among multiple latent attributes (Hansen, 2013; Zhan et al., 2019). The HMM/ANN model proposed in this study is a further enrichment of longitudinal CDMs. Meanwhile, the second approach is consistent with the overall idea of establishing the HMM/ANN model to realize the longitudinal tracking of the cognitive skills of students in this study. Notably, both utilized the processing capability of HMM for time-series changes and integrated the diagnostic classification model.

Compared with the MIRT method, the combination of these two models can achieve fine-grained longitudinal tracking of students’ cognitive skills. Meanwhile, the method based on MIRT can only obtain how students develop in a single ability. However, students with the same original score may master different skills. Compared with combining HMM with traditional CDMs, the HMM/ANN model proposed in this study has an advantage in the classification accuracy of cognitive skills. As mentioned before, traditional CDM is based on the framework of IRT, and the accuracy of model parameter estimation and classification will be affected when the data are unable to meet the strong hypothesis of IRT or the sample size is small. Because it is not necessary for ANN to perform parameter estimation, it can also obtain a higher classification accuracy when the data do not meet the assumptions of unidimension, local independence, and monotonicity or when the samples are small (Cao, 2009; de La Torre et al., 2010; Chen et al., 2013). This study also supports this. Moreover, the third method takes the problem of local item dependence into account and overcomes the defects of its application in the real educational situation to some extent. However, it still used in the parameter estimation method. In contrast, the HMM/ANN model is non-linear and is not affected by the characteristics of sample distribution and data types. It does not need to meet the strong assumption of IRT or require parameter estimation and is relatively less affected by the sample size. Consequently, the HMM/ANN model is more suitable for data collected in real educational situations and does not need a large scale to obtain good results, and it can also effectively track the changes in the cognitive skills of students in the context of small sample size in schools or classes.

In addition, considering how well the model matches the data, the HMM/ANN model may not always be more powerful than other longitudinal CDMs. For example, based on the DINA model to generate data, the DINA model is used to estimate the model so that the model truly fits the data, and the result will be more powerful than the ANN model; however, when the model and the data are misfit, the advantages of ANN are obvious (Cui et al., 2016).

### The Classification Accuracy of SSOM in the HMM/ANN Model Is Affected by Some Factors

The SSOM applied in this study can accurately classify cognitive skills when the test examines three attributes. However, when multiple attributes are incorporated in the test, SSOM demonstrates a lower classification accuracy. This result can be explained because, in the process of algorithm operation, it does not establish a direct mapping relationship between the students’ response (input data) and the mastery of each skill (output data). It instead initially obtains the attribute mastery patterns based on the response to the input, and finally outputs the mastery of each skill. As the number of attributes increases, the total number of attribute mastery patterns increases exponentially. When the test examined only three attributes, there were a total of 2^3^ = 8 attribute mastery patterns, and each student was classified into one of the eight attribute mastery patterns. Meanwhile, when the number of attributes increases to six, 2^6^ = 64 attribute mastery patterns are generated. To classify students into the correct attribute mastery patterns, it is necessary to identify the students’ mastery and non-mastery of each of the six attributes. If one attribute is misclassified, the entire attribute mastery pattern is misclassified as well (Cui et al., 2016).

The classification accuracy of SSOM is also affected by other factors. The greater the number of items and the higher the quality of those items, the higher the classification accuracy of SSOM. The items with low discrimination are not suitable for distinguishing the students’ mastery of skills, and it also affects the accuracy of the estimation of attributing mastery (Roussos et al., 2005). The sample size has little influence on the classification accuracy of SSOM. Notably, SSOM’s classification results are relatively accurate both in empirical as well as in simulation studies, with sample sizes of 200, 500, or 1,000. Despite so, however, SSOM’s classification accuracy is slightly lower than that of 500 or 1,000 samples under the condition of 20 items and 200 samples. However, when the number of questions increased, the classification accuracy of SSOM was relatively consistent under the condition of three sample sizes. Since the ANN does not need parameter estimation, the sample size has little influence on the accuracy of its classification (Gierl et al., 2008; Shu et al., 2013). Furthermore, SSOM is essentially a clustering algorithm, so it is more affected by the number of categories.

The advantage of applying the HMM/ANN model in a real educational situation is that its measurement model is ANN, which does not require parameter estimation under the framework of IRT. When the data cannot meet the hypothesis of IRT and the sample size is small, ANN can assist in obtaining accurate classification results, which makes CDA more widely applied in daily teaching.

Teachers can make use of this model to understand the development of students’ cognitive skills, understand the advantages and disadvantages of students’ skill mastery, and adjust teaching strategies or teaching priorities in a timely manner. However, the application of this longitudinal cognitive diagnosis model should be conducted cautiously. As the results highlight, ANN does not perform well under all conditions. To obtain a more accurate classification and accurately track the development of students’ cognitive skills, teachers and educators should examine the appropriate number of attributes and design high-quality items when applying the HMM/ANN model so as to ensure the accurate classification of cognitive skills.

## Conclusion, Limitations, and Further Study

This study constructs a new theoretical model of longitudinal CDA, which combines HMM with ANN, making full use of HMM’s advantages to process time series information and the advantages of ANN to process non-linear information to realize the tracking of cognitive skills. This is a useful exploration of the longitudinal CDM and will help promote the technical development of the longitudinal CDA. The purpose of this study was to verify the effectiveness of the proposed HMM/ANN model in longitudinal cognitive diagnostic analysis under different conditions. The results of the simulation and the empirical studies illustrate that the HMM/ANN model can accurately classify cognitive skills and track the development of students’ cognitive skills. Consequently, it is reasonable to use the developed model to track the development of students’ cognitive skills. Additionally, the classification accuracy of SSOM is better when the number of attributes is low, the number of items is high, and the quality of items is better. Furthermore, the sample size has a slight influence on the classification accuracy of SSOM.

The simulation results demonstrate that the HMM/ANN model has a high correct transition rate under various simulation conditions. When the cognitive skills examined were relatively small, the correct transition rate of HMM/ANN was consistent with the classification accuracy of ANN. When the classification accuracy of ANN was low, the correct transition rate was also relatively low, which is consistent with previous studies (Kaya and Leite, 2017). When a relatively large number of skills were examined, the correct transition rate of the HMM/ANN model was overestimated. Meanwhile, as skills increased, the attribute mastery patterns increased exponentially, forming a larger transformation probability matrix of the attribute mastery pattern. However, the students’ attribute mastery pattern is usually concentrated in several categories, so the correct transformation probability has been overestimated. In the empirical study, the HMM/ANN model can assist in obtaining information concerning the students’ mastery of each reading skill as well as with the development of reading skills.

This study is a new attempt in using ANN in longitudinal CDA, and there are some limitations and prospects. First, when the number of attributes examined is large, the ANN still cannot achieve better classification results. In future studies, the diagnostic classification model should be further optimized so as to explore the CDMs applicable to classify more attributes. Second, the development of students’ mastery of skills in the empirical study does not explain whether the development is caused by teachers’ instruction or if it is from the students’ natural development. However, this does not affect the results of this study because the focus is to verify whether the proposed HMM/ANN model can accurately track the development of students’ cognitive skills. Moreover, it is necessary for the HMM/ANN model to be verified in more educational contexts, so the application of other types of ANNs in CDA can be further tested. Finally, this study did not compare the HMM/ANN model with more longitudinal CDMs because we are more inclined to provide an alternative model rather than to judge whether HMM/ANN has an absolute advantage. In the future, we will further compare this model with a more powerful model.

## Data Availability Statement

The raw data supporting the conclusions of this article will be made available by the authors, without undue reservation.

## Ethics Statement

The studies involving human participants were reviewed and approved by Institutional Review Board of the Faculty of Psychology, BNU. Written informed consent to participate in this study was provided by the participants’ legal guardian/next of kin.

## Author Contributions

HW conceived and designed the study, collected the data, and helped in performing the analysis with constructive discussions. YL and NZ performed the data analyses and wrote the manuscript. All authors contributed to the article and approved the submitted version.

## Conflict of Interest

The authors declare that the research was conducted in the absence of any commercial or financial relationships that could be construed as a potential conflict of interest.
